# Comparative Study of Curvature Sensing Mediated by F-BAR and an Intrinsically Disordered Region of FBP17

**DOI:** 10.1016/j.isci.2020.101712

**Published:** 2020-10-20

**Authors:** Maohan Su, Yinyin Zhuang, Xinwen Miao, Yongpeng Zeng, Weibo Gao, Wenting Zhao, Min Wu

**Affiliations:** 1 Department of Cell Biology, Yale University School of Medicine, 333 Cedar Street, New Haven, CT 06520-8002, USA; 2 Centre for BioImaging Sciences, Mechanobiology Institute, Department of Biological Sciences, National University of Singapore, Singapore, 117411; 3 School of Chemical and Biomedical Engineering, Nanyang Technological University, Singapore, 637457; 4 School of Physics and Mathematical Science, Nanyang Technological University, Singapore, 637371

**Keywords:** Biological Sciences, Cell Biology, Biophysics

## Abstract

Membrane curvature has emerged as an intriguing physical principle underlying biological signaling and membrane trafficking. The CIP4/FBP17/Toca-1 F-BAR subfamily is unique in the BAR family because its structurally folded F-BAR domain does not contain any hydrophobic motifs that insert into membrane. Although widely assumed so, whether the banana-shaped F-BAR domain alone can sense curvature has never been experimentally demonstrated. Using a nanobar-supported lipid bilayer system, we found that the F-BAR domain of FBP17 displayed minimal curvature sensing *in vitro*. In comparison, an alternatively spliced intrinsically disordered region (IDR) adjacent to the F-BAR domain has the membrane curvature-sensing ability greatly exceeding that of F-BAR domain alone. In living cells, the presence of the IDR delayed the recruitment of FBP17 in curvature-coupled cortical waves. Collectively, we propose that contrary to the common belief, FBP17's curvature-sensing capability largely originates from IDR, and not the F-BAR domain alone.

## Highlights

• An intrinsically disordered region (IDR) of FBP17 senses membrane curvature
• The curvature sensing ability of IDR greatly exceeds the F-BAR domain of FBP17
• IDR modulates the phase of FBP17 during wave propagation in live cells

## Graphical Abstract

**Figure:**
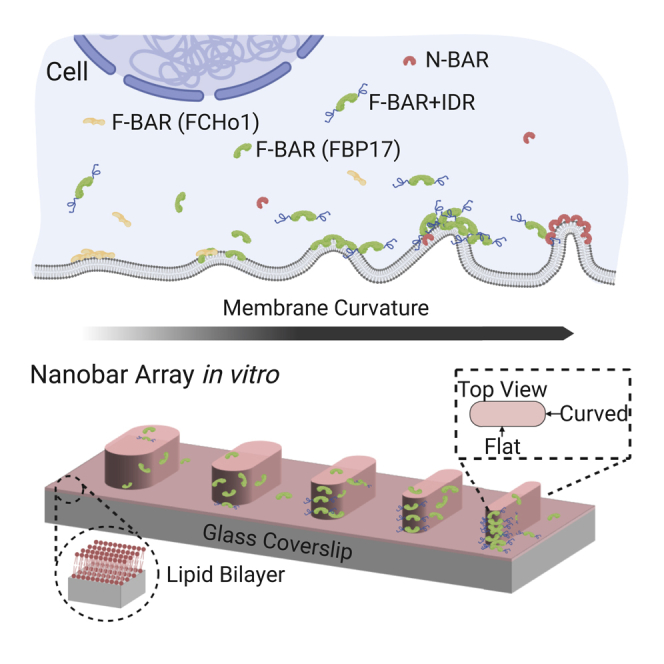

## Introduction

Membrane curvature in cells needs to be dynamically generated and sensed by peripheral membrane proteins in biological processes involving membrane deformation. In principle, curvature-sensing proteins should be more prevalent than generators because some of the sensors that are too flexible to bend the membrane may fail to generate curvature (Zimmerberg and Kozlov, 2006). In reality, numerous proteins have been reported to deform cellular membranes but much fewer proteins have been shown to sense membrane curvature (Antonny, 2011). Although curvature generation and sensing have been proposed as behaviors by the same proteins at different concentration regimes (Simunovic et al., 2018; Suetsugu and Gautreau, 2012), whether proteins generating curvature are by default curvature sensors remains unknown (Madsen et al., 2010).

The BAR (Bin/Amphiphysin/Rvs) domain-containing superfamily proteins share banana-shaped BAR domains that can bind to membrane via their concave faces (Farsad and De Camilli, 2003; Kozlov et al., 2014; McMahon and Gallop, 2005). Because of their shapes (Frost et al., 2009), they are intuitively assumed as both curvature generators and sensors. Many BAR proteins also contain amphipathic helices. Some of these helices are essential for curvature sensing (Bhatia et al., 2009), whereas others seems to be disposable (Peter, 2004; Simunovic et al., 2016). Therefore, whether a scaffolding mechanism alone is sufficient to sense curvature remains controversial (Bhatia et al., 2010; Simunovic et al., 2015). F-BAR proteins of FBP17/CIP4/Toca sub-family are intriguing examples to study the contribution of scaffolding mechanisms in curvature sensing for their lack of amphipathic helices or hydrophobic insertion region present in other BAR domains (Qualmann et al., 2011). Previous experimental evidences on the curvature sensing of the F-BAR domain in this sub-family are lacking. Full length proteins of Toca1 and FBP17 were shown to prefer larger liposomes of 1000 nm than smaller ones (Takano et al., 2008). In addition to the FBP17/CIP4/Toca sub-family, other F-BAR domains of Cdc15 and FCHo2 showed no curvature preference (Henne et al., 2007; McDonald et al., 2016), whereas F-BAR of syndapin1 (containing hydrophobic insertion motifs) preferred smaller liposomes (<100 nm diameter) (Ramesh et al., 2013). These evidences suggest that scaffolding mechanism may not be necessary or even sufficient for curvature sensing in BAR proteins.

Intrinsically disordered proteins can also sense curvature. They either become folded upon binding to membrane, such as α-synuclein (Davidson et al., 1998; Pranke et al., 2011) and amphipathic lipid packing sensor (ALPS) motifs (Bigay et al., 2005), or remain disordered on membrane such as MARCKS-ED (Morton et al., 2013). Intrinsically disordered regions (IDRs) from endocytic proteins such as AP180 or Amphiphysin can also sense curvature when they are artificially tethered to the membrane (Zeno et al., 2018, 2019). Of particular interest to this study, the IDR of F-BAR protein Fer has been shown to sense curvature using liposome binding assay (Yamamoto et al., 2018).

One fascinating feature of BAR superfamily proteins is the wide spectrum of curvature in the shape they display, apparent from both the crystal structures of BAR domains and the diameters of the tubules they generate *in vitro*. We still understand little about why different types of curvatures are necessary for cells. Although *in vitro* systems are ideal to address the question of sufficiency in curvature sensing or generating, live cell assays are needed to address the question of necessity, with a caveat of mixing curvature generation and sensing (Suetsugu et al., 2014). Our recent work on cortical waves provides a unique opportunity. In mast cells, many cortical proteins such as active Cdc42 and actin are assembled into traveling waves or oscillatory standing waves (Wu et al., 2013). F-BAR domain proteins FBP17 and CIP4 are essential for cortical wave formation because their double knockdown (DKD) leads to the elimination of such patterns (Wu et al., 2018). As these cytosolic proteins cycle on and off the membrane, membrane curvature develops with F-BAR proteins waves. Importantly, in DKD cells, only FBP17 and mutants with slightly shallower curvature preferences (F-BAR^K66E^), but not those with higher curvature (F-BAR^K166A^), can rescue wave formation. Hence we proposed a mechanochemical feedback model showing that curvature-dependent recruitment of F-BAR proteins was essential for wave propagation, and this provides a physiologically relevant context to address the unique role of F-BAR domains (Wu et al., 2018).

In this work, we employed both the cortical wave system and *in vitro* lipid-bilayer-coated nanobar approach to characterize curvature sensing of FBP17. In propagating cortical waves, we showed that the phase of FCHo, FBP17, and endophilin recruitment to the traveling waves is primarily governed by their membrane-binding domains and can be predicted from their curvature preference. We found that the phases of two FBP17 isoforms (FBP17S and FBP17L) consistently differ, and a differentially spliced IDR modulates protein's phase in waves. We then developed an *in vitro* nanobar-supported lipid bilayer (SLB) system with defined curvature to test curvature sensing, based on a previous study (Zhao et al., 2017). Using purified proteins, we showed that the F-BAR domain of FBP17 displayed positive but weak curvature sensitivity. However, more dominant ability on curvature sensing appears to originate from this previously uncharacterized IDR following F-BAR domain.

## Results

### Phases of Protein Recruitment to the Traveling Waves Correlate with Curvature Progression

**Figure 1:**
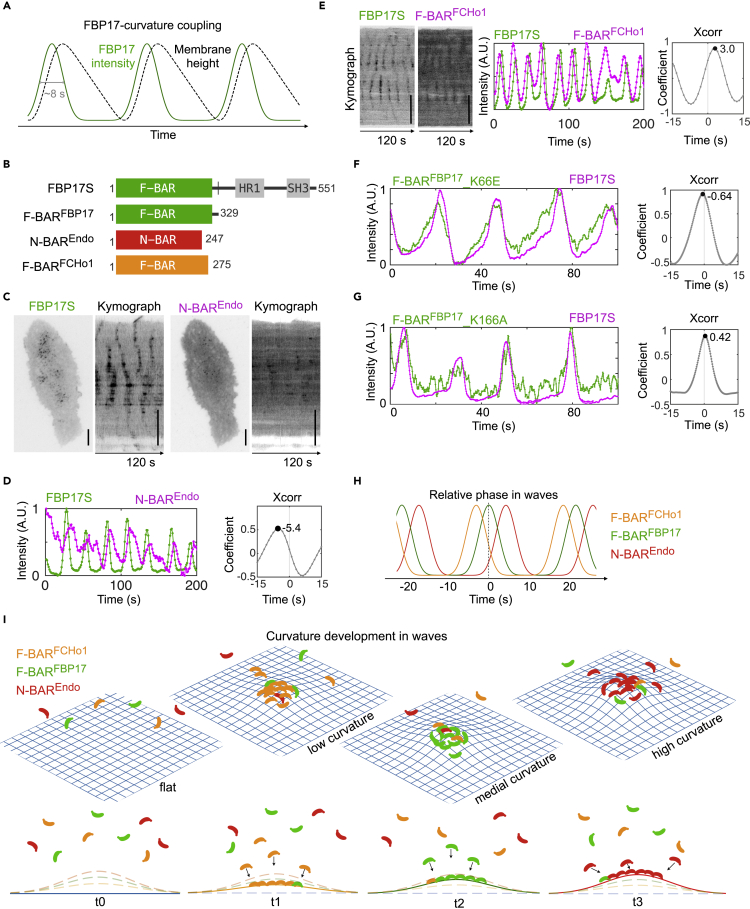
Dynamic Recruitment of FBP17, F-BAR, and N-BAR to Traveling Waves (A) Diagram of FBP17 versus membrane height change in traveling waves. The lifetime of FBP17 per cycle is about 8 s. (B) Diagram of constructs used: FBP17S, F-BAR^FBP17^(FBP17 aa 1–329), F-BAR^FCHo1^(FCHo1 aa 1–275), and N-BAR^Endo^ (Endophilin-1 aa 1–247). (C) TIRF micrographs and kymographs of traveling waves of a representative RBL cell co-overexpressing GFP-FBP17S (left) and N-BAR^Endo^-mCherry (right). Scale bar, 10 μm. (D) Left: Intensity profile of GFP-FBP17S (green) and N-BAR^Endo^-mCherry (magenta) of the same ~2 × 2-μm^2^ region of interest (ROI) inside the cell in (C). Right: cross-correlation (Xcorr) of the two signals in left shows the phase shift of waves of GFP-FBP17S relative to that of N-BAR^Endo^ -mCherry. Time resolution: 1.0 s. (E) Kymographs (left), intensity profile of the ~2 × 2-μm^2^ ROI (middle), and cross-correlation (right) of waves of F-BAR^FCHo1^-GFP (green) relative to that of mCherry-FBP17S (magenta). Scale bar, 10 μm. Time resolution: 1.0 s. (F) Intensity profile (left) and cross-correlation (right) of F-BAR^FBP17^_K66E-GFP relative to mCherry-FBP17S. Time resolution: 0.21 s. (G) Same as (F), but with F-BAR^FBP17^_K166A-GFP. (H) Diagram showing phase shift in waves of F-BAR^FCHo1^ and N-BAR^Endo^ compared with F-BAR^FBP17^. Colors denote different protein domains. (I) Top: Summary diagram of continuous curvature development in traveling waves shown at four exemplary stages. From a flat membrane (t_0_ when minimal protein-membrane binding), waves are initiated (t_1,_ first time point) with shallow curvature, which F-BAR^FCHo1^ prefers and binds to, and continuously develop to higher and higher curvature, which attracts F-BAR^FBP17^ (t_2_) and later N-BAR^Endo^ (t_3_) the most. Plasma membrane is shown as blue mesh. Bottom: 2D cross section of the top. Dashed lines denote the membrane at other time points. Colors denote different protein domains.

We previously showed that wave formation in cells was coupled with changes in membrane curvature as illustrated in Figure 1A, and our mechanochemical feedback model predicted that curvature (as inferred by membrane height) was critical for fast wave propagation (Wu et al., 2018). As the changes in membrane curvature must be continuous, we set out to test to what extent the timing of the peak recruitment of curvature-generating proteins, i.e., the phase of the protein in waves, is dominated by their lipid binding and could correlate with their curvature preferences measured *in vitro*. We first imaged FBP17S (the commonly used short isoform of FBP17 corresponding to rat Rapostlin) and N-BAR domain only (amino acids [aa] 1–247) from Endophilin-1 (N-BAR^Endo^) (Figure 1B), which has the ability to induce membrane tubules with higher curvature (Mim et al., 2012) than the F-BAR domain from FBP17 (Frost et al., 2008). By co-overexpressing them in RBL cells, we found that N-BAR^Endo^ could assemble as dynamic puncta in the form of traveling waves together with FBP17S (Figure 1C), and its phase lagged FBP17S for approximately 5 s (Figure 1D). N-BAR^Endo^ was in the same phase as full-length Endophilin (Figure S1A). When we replaced the F-BAR domain of FBP17S with N-BAR^Endo^, this chimeric protein appeared in waves in a phase that is different from wild-type FBP17S but is the same as full-length Endophilin (Figure S1B). These data suggest that the timing of FBP17 recruitment is determined by its membrane-interacting domains and can be modulated by changing its membrane interaction. We also tested the N-BAR domain (aa 1–236) from Amphiphysin-1 (N-BAR^Amph^), which shares similar intrinsic curvature with N-BAR^Endo^ (Mim et al., 2012; Peter, 2004). N-BAR^Amph^ also formed dynamic puncta as waves and its phase lagged FBP17S by 5 s (Figure S1C), indicating that this phase shift may be dictated by common property of N-BARs.

While membrane curvature is likely continuously generated, sequential recruitments of curvature-sensing proteins can only happen if membrane curvature is the rate-limiting step for their recruitment and the rate of membrane bending is sufficiently slow to allow curvature sensing. We further tested our curvature progression model by examining proteins showing even shallower curvature preferences than F-BAR^FBP17^. The F-BAR domain (aa 1–275) from FCHo1 (F-BAR^FCHo1^) induces membrane tubules with larger diameter than F-BAR^FBP17^ (Henne et al., 2007). We found that it was recruited to membrane waves 3 s before FBP17S (Figure 1E). This relative timing was independent of the fluorescent tags used (comparing Figures 1E, S1D, and S1E) and imaging acquisition intervals (Figures S1D and S1E). In summary, the sequential recruitments of these domains are well-correlated with the timing of each corresponding full-length protein in waves (Figure 1H) (Yang et al., 2017).

To test whether the phases of these waves could reveal even smaller differences in curvature, we compared the effects of point mutations K66E and K166A in F-BAR^FBP17^. K66E mutant (F-BAR^K66E^) generates wider tubules *in vitro*, whereas K166A mutant (F-BAR^K166A^) generates narrower tubules than wild-type F-BAR^FBP17^ (Frost et al., 2008), but both to a lesser degree than F-BAR^FCHo1^ or N-BAR. Using fast stream acquisition mode at 5Hz (0.2 s/frame), we found that F-BAR^K66E^ shifted the phase ahead of FBP17S by about 0.6 s (three frames of imaging) (Figure 1F) and F-BAR^K166A^ delayed the phase by about 0.4 s (Figure 1G). Collectively, the data are consistent with our hypothesis that plasma membrane in waves gradually develops from no to low curvature, and maximal protein binding of curvature-sensing proteins occurs at different stages of wave formation according to their curvature preferences, as illustrated in Figure 1I.

### Two Isoforms of FBP17 Differ in Their Phases of Recruitment to Waves

**Figure 2:**
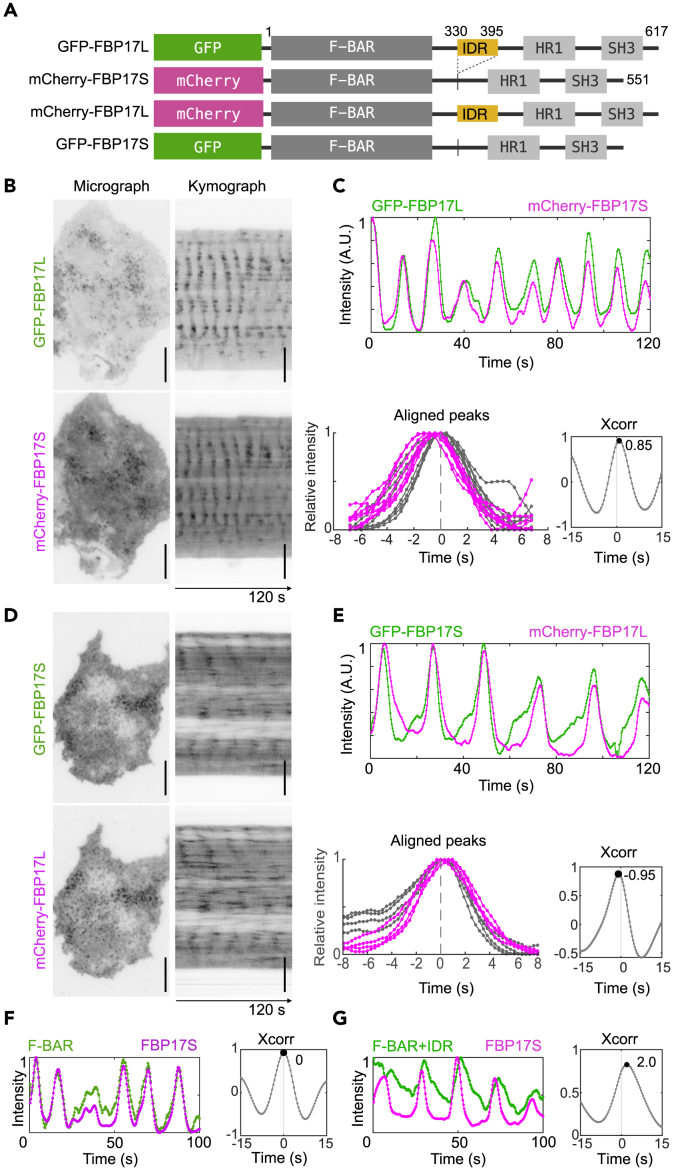
Phase Shifts between FBP17 Isoforms in Traveling Waves (A) Diagram of domain structures of long isoform FBP17 (FBP17L) and short isoform (FBP17S) with fluorescence tags fused at the N terminus. The IDR is shown in yellow. **(B)** Images (**left**) and kymographs (**right**) of co-overexpressed GFP-FBP17L and mCherry-FBP17S in RBL cells. **(C) Top**: Intensity profile of GFP-FBP17L (**green**) and mCherry-FBP17S (**magenta**) channels. **Bottom left**: aligned peaks of the green channel (shown in grey) from the **top** plots. Signal in mCherry channel (**magenta**) was plotted based on the same alignment in GFP channel. **Bottom right:** Cross-correlation of GFP relative to mCherry channel. **(D)** Images (**left**) and kymographs (**right**) of co-overexpressed FBP17 GFP-FBP17S and mCherry-FBP17L in RBL cells. **(E) Top**: Intensity profile of GFP-FBP17S (**green**) and mCherry-FBP17L (**magenta**) channels. **Bottom left**: aligned peaks of the green channel (shown in grey) from the **top** plots. Signal in mCherry channel (**magenta**) was plotted based on the same alignment in GFP channel. **Bottom right:** Cross-correlation of GFP relative to mCherry channel. (F and G) Intensity profile (left), and cross-correlation (right) of F-BAR^FBP17^-GFP (F) and F-BAR+IDR-GFP (G) relative to FBP17S-mCherry. Scale bar: 10 μm in (B and D). Time resolution: 0.42 s (B and C), 0.32 s (D and E), 0.40 s (F and G).

FBP17 has two major isoforms. The short one is referred as FBP17S and the long one as FBP17L throughout this paper (Kakimoto et al., 2004) (Figure 2A). The only difference between these two isoforms is the presence of 66 amino acids starting at the 330^th^ from the N terminus of the long isoform. We co-expressed FBP17S and FBP17L in the same cell and found that both isoforms were recruited to the waves (Figure 2B). Interestingly, we consistently found a phase difference between them, with FBP17S preceding FBP17L by 0.8–1.0 s, as quantified by cross-correlation analysis (Figure 2C). We also confirmed that the relative phase differences between these two isoforms did not depend on which fluorescent tags were used and the order of sequential two-color image acquisition (Figures 2D and 2E) or whether the fluorescent tags were at the N or C terminus (Figures S2A and S2B).

To test whether the observed phase differences were due to the membrane-binding differences between FBP17S and FBP17L, or more complex mechanisms such as conformational changes involving domains at the C terminus and other protein recruitment, we deleted the C-terminal aa 330–551 and aa 396–617 (they are the same protein sequence containing HR1 and SH3 domains) from FBP17S and FBP17L, respectively. We co-expressed the remaining F-BAR domain (aa 1–329) with FBP17S. The F-BAR domain participated in the waves at the exact same phase of FBP17S (Figure 2F), suggesting that the phase of FBP17S was set by the F-BAR domain, but not its C-terminal region. However, aa 1–395 from FBP17L (F-BAR + IDR) (Figure S2A) was recruited about 2 s later than FBP17S (Figure 2G). The phase lag was also independent of the time interval we chose, as long as it was less than half of the phase lag (Figures S2C and S2D). We thus concluded that the observed phase differences were due to the presence of the aa 330–395 after the F-BAR domain in FBP17L.

### FBP17L Contains Conserved Alternatively Spliced IDR

**Figure 3:**
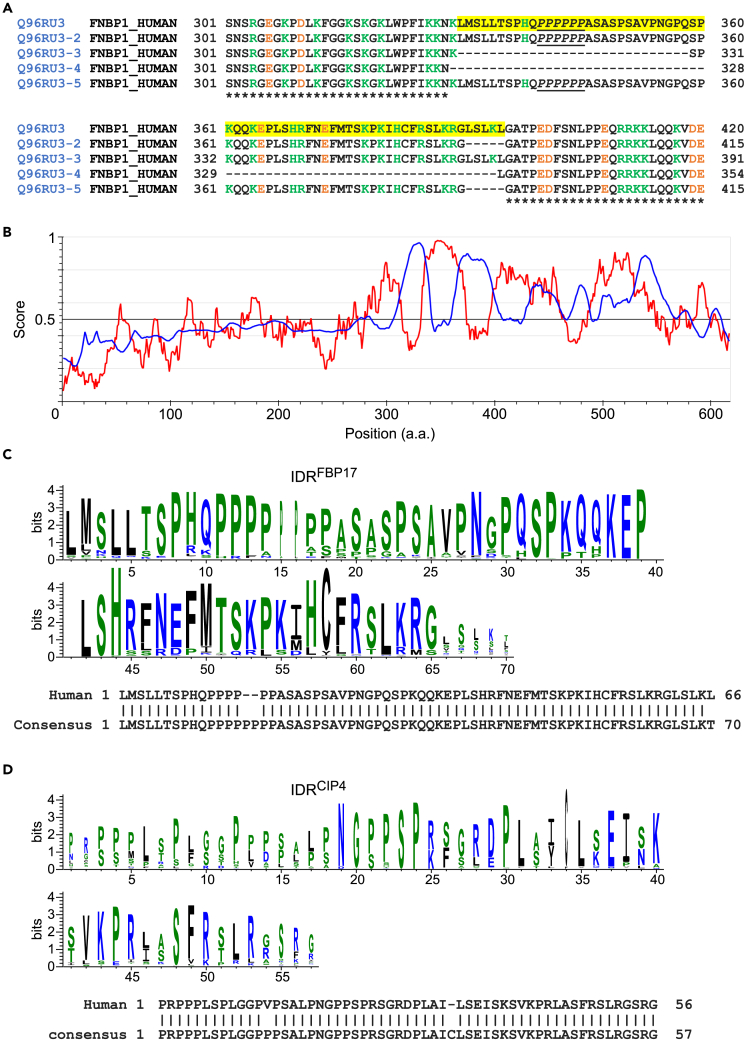
FBP17L Contains a Conserved IDR (A) Aligned alternatively spliced isoforms of human FBP17. The ∗ is shown below at each position when the residue is conserved. Negative and positive amino acids are shown in orange and green, respectively. The IDR is highlighted in yellow. PolyP region is underlined. (B) Intrinsic disorder prediction of human FBP17L by IUPred2A (red), and protein-binding prediction by ANCHOR2 (blue). (C) Top: WebLogo of the IDR of FBP17 from all 299 species that have FBP17. Blue: hydrophilic amino acid. Green: neutral amino acid. Black: hydrophobic amino acid. Bottom: Sequence alignment of the IDR from human FBP17L and the consensus sequence from WebLogo 3. (D) Top: WebLogo of the IDR of CIP4 from all 183 species that have CIP4. Bottom: Sequence alignment of the IDR from human CIP4 and the consensus sequence from WebLogo 3.

The shorter isoform FBP17S lacked the region aa 330–395 present in FBP17L. This region was alternatively spliced in FBP17 (Figure 3A). It was predicted to be intrinsically disordered and low in protein binding (Figure 3B). In this article, we refer to this region of human FBP17L as IDR^FBP17L^. Interestingly, unlike many other IDRs (Van Der Lee et al., 2014), the amino acid sequence of the IDR^FBP17L^ was evolutionarily conserved. We found that the protein FBP17 appeared in the phyla Chordata and Arthropoda according to GenBank database, and the exact sequence of the IDR of human FBP17L was found in the clade Amniota comprising the reptiles, birds, and mammals (see Transparent Methods). To see the sequence conservation, we generated the sequence logo (Crooks et al., 2004) from all FBP17 sequences (Figure 3C). At each position, the overall height (entropy) depicted the information content (in bits) indicative of the sequence conservation, whereas the width (weight) was proportional to the fraction of existence. IDR^FBP17L^ had few mutations as shown by the alignment between the IDR of human FBP17L and consensus between IDRs of FBP17 from all species. For comparison, the paralog of FBP17, CIP4 (TRIP10), shared a sequence similar to FBP17 (71% positive for full-length and 66% for the corresponding IDR) (Figure S3A) but the sequence logo of its IDR (IDR^CIP4^) (Figure 3D) was lower in entropy and weight (P < 0.0001, Mann-Whitney U test) (Figure S3C), indicating that the IDR^FBP17L^ was more evolutionarily conserved than IDR^CIP4^. The other paralog Toca-1 (FNBP1L) was also analyzed, and its IDR was more conserved than IDR^CIP4^, but its weight was less than IDR^FBP17L^, indicating a higher likelihood of being spliced (Figures S3B and S3C).

### IDR of FBP17L Directly Senses Membrane Curvature *In Vitro*

**Figure 4:**
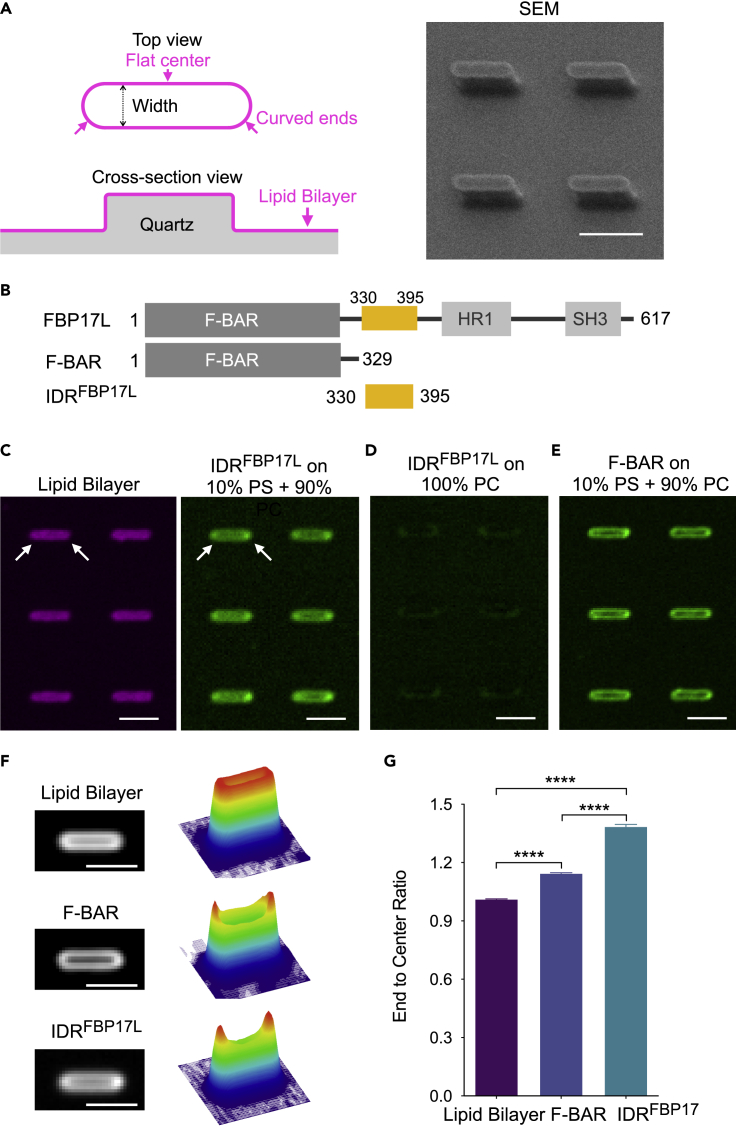
IDR of FBP17L Senses Membrane Curvature *In Vitro* (A) Schematic illustration of lipid-bilayer-coated nanobar structure (left) and the scanning electron micrographic image of fabricated nanobar array (right). Scale bar, 2 μm. (B) Diagram of domain structures of FBP17L and its truncations: F-BAR (aa 1–329) and IDR^FBP17L^ (aa 330–395). (C) Confocal images of lipid bilayer (10% PS: 89.5% PC: 0.5% Texas Red DHPE) on top of nanobars of 300 nm width (left) and 16 μM Alexa Fluor 647-IDR^FBP17L^ on the bilayer (right). Arrowheads indicate two nanobar ends. Scale bar, 2 μm. (D) 16 μM Alexa Fluor 647-IDR^FBP17L^ on 100% PC bilayer-coated nanobars of 300 nm width. Image contrast was the same as the right image in (C). Scale bar, 2 μm. (E) Confocal images of 16 μM Alexa Fluor 488- F-BAR coated on 10% PS in PC bilayer-coated nanobars of 300 nm width. Scale bar, 2 μm. (F) Average images and the corresponding 3D surface plot of lipid bilayer, F-BAR, and IDR^FBP17L^ on over 100 nanobars of 300 nm width. Scale bar, 2 μm. (G) End to center ratio of lipid bilayer, F-BAR, and IDR^FBP17L^ on 200 nanobars of 300 nm width (n = 3 experiments per protein). Unpaired t test with Welch's correction: ∗∗∗∗p < 0.0001. Data are represented as mean ± SEM (standard error).

Although our results in live cells suggest a possible role of the IDR^FBP17L^ in curvature sensing, it will be difficult to dissect its direct interaction with curved membrane without removing other cellular components in proximity that can potentially bind to the IDR^FBP17L^ and facilitate its membrane binding. We therefore developed an *in vitro* system based on the recently established nanobar platform to directly test curvature sensing of the IDR and F-BAR from human FBP17L (Li et al., 2019; Zhao et al., 2017). As illustrated in Figure 4A left, one nanobar contained two half-circled ends with curvature diameter defined by the bar width, whereas the flat middle served as local zero-curvature control. The scanning electron micrograph shows two arrays of vertically aligned nanobars 300 nm in width, 2 μm in length, and 600 nm in height fabricated via electron beam lithography (Figure 4A right). To study the differential binding of proteins on curved membrane, we coated a SLB consisting of 10% brain phosphatidylserine (PS) and 90% egg phosphatidylcholine (PC) on the nanobar arrays (see Transparent Methods). The SLB covered both the nanobars and surrounding flat area. Neither synthesized IDR^FBP17L^ nor purified F-BAR contained additional tags for membrane binding but could bind to the nanobar-based SLB (Figure 4B). Unlike uniform lipid signal throughout the contour of nanobars (Figure 4C, left), IDR^FBP17L^ showed stronger fluorescent signals at nanobar ends in comparison with nanobar center, reporting a higher protein density at curved membrane sites (Figure 4C, right). It is noteworthy that on neutrally charged nanobar-SLB with only PC but no PS, IDR^FBP17L^ signal was ∼90% weaker than that on negatively charged membrane with PS (Figures 4D and S4), suggesting that the direct membrane binding of IDR^FBP17L^ was charge dependent. F-BAR also preferentially binds to curved nanobar ends with negatively charged lipids, but to a lesser extent than IDR^FBP17L^ (Figure 4E). The difference in curvature preferences between IDR^FBP17L^ and F-BAR was more detectable in images averaged over 100 nanobars (Figure 4F). Using the ratio of fluorescence intensities of nanobar end to center as an indicator of curvature preference (Figure 4G), we observed significant differences between IDR^FBP17L^ and F-BAR (ratio of 1.385 ± 0.011 and 1.144 ± 0.004 respectively, mean ± SEM [standard error], unpaired t test, p < 0.0001), with both of them giving higher ratios than lipid bilayer (1.012 ± 0.001, mean ± SEM, unpaired t test, p < 0.0001). Collectively, these data suggest that the IDR or F-BAR from human FBP17L is capable of binding to negatively charged lipid membrane and sensing curved membrane directly.

**Figure 5:**
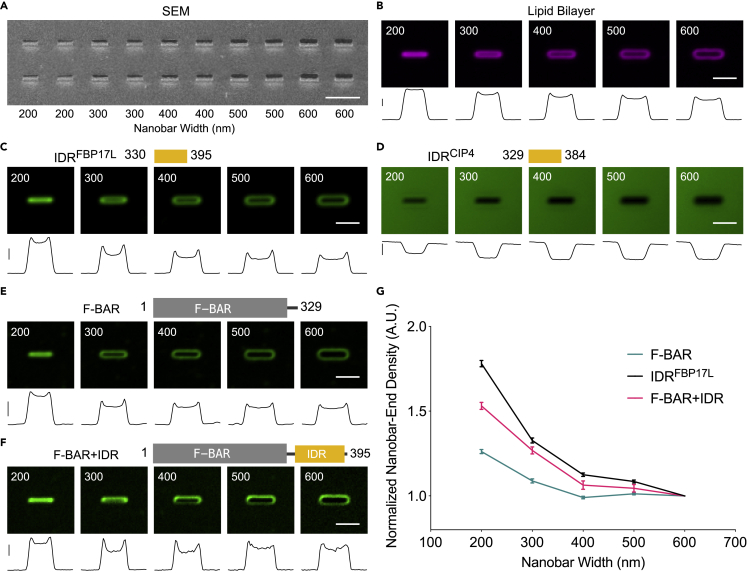
Curvature Sensing of IDR^FBP17L^, F-BAR, and F-BAR + IDR on Nanobar Array (A) SEM image of nanobar arrays in width gradient from 200 to 600 nm with 100-nm step. Every two columns of the arrays are the same size as indicated. The height of every nanobar is 600 nm. Scale bar, 5 μm. (B–F) Diagrams (top) and averaged images (middle) of lipid bilayer (B), 16 μM IDR^FBP17L^ (C), 25 μM IDR^CIP4^ (D), 16 μM F-BAR (E), and 16 μM F-BAR + IDR (F) on nanobars with width from 200 nm to 600 nm. Each image was averaged from over 60 nanobars. Scale bar, 2 μm. Bottom: Intensity profiles along nanobar of each averaged image. Scale bars: 10,000, 2,000, 10, 2,000, 5,000 a.u. in (B) to (F). (G) Normalized nanobar-end density of FBP17 F-BAR, IDR, and F-BAR + IDR based on their corresponding lipid bilayer intensity. Each point represents mean ± SEM from over 60 (n = 3 experiments per protein).

To examine the range of membrane curvatures that the IDR^FBP17L^ senses, we employed the nanobar array with a series of widths from 200 nm to 600 nm in 100-nm increments (Figure 5A). Compared with the homogeneous lipid coating on all different-sized nanobars (Figure 5B), IDR^FBP17L^ showed higher signals at the nanobar ends as the diameter of the bar-ends curvature decreased below 400 nm (Figure 5C). In comparison, the corresponding IDR^CIP4^ had no detectable binding to the lipid bilayer at any of the sizes tested (Figure 5D), suggesting a unique role of IDR^FBP17L^ in curvature sensing. Similar to IDR^FBP17L^, F-BAR and F-BAR + IDR also showed increased signals at bar ends (Figures 5E and 5F). To account for the surface area changes across nanobars of different sizes, we measured the protein density at nanobar ends, i.e., the bar-end intensity of each protein per unit of the fluorescent lipid's intensity accordingly. All three proteins showed preferential binding to higher membrane curvatures (Figure 5G), with IDR^FBP17L^ showing the highest density, F-BAR the lowest, and F-BAR + IDR at an intermediate level.

### IDR^FBP17L^ Has Faster Dissociation Rate *In Vitro* Than F-BAR Domain

**Figure 6:**
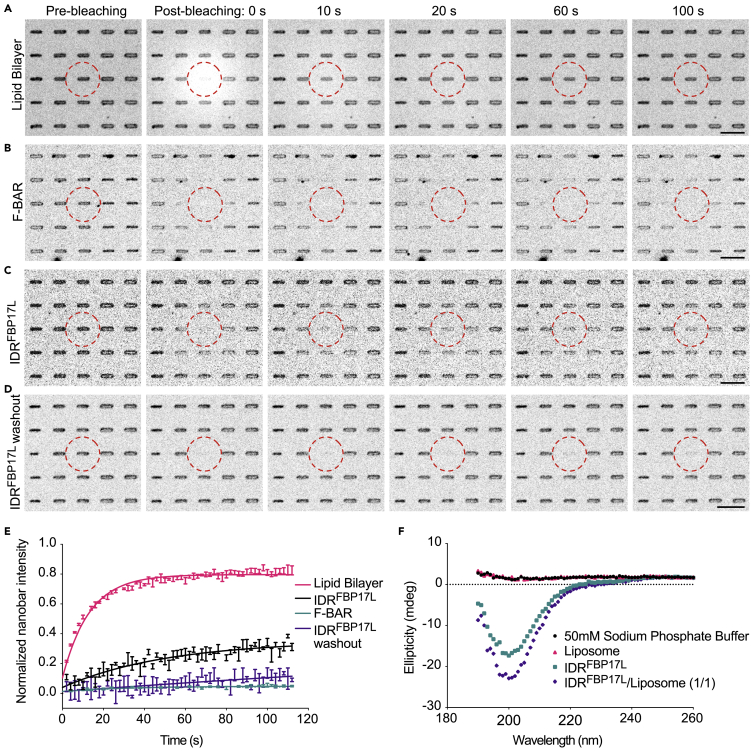
Membrane Binding Dynamics of IDR^FBP17L^ and F-BAR (A–D) Montages of confocal FRAP test on lipid bilayer (A), F-BAR (B), IDR^FBP17L^ (C), and IDR^FBP17L^ washed out (D) on nanobar with 300 nm width in 2 min. Red dashed circles indicate the bleaching area. Scale bar, 5 μm. (E) Normalized intensity plot of lipid bilayer, F-BAR, IDR^FBP17L^, and IDR^FBP17L^ washed out within nanobar area (21 × 7 pixels) from FRAP measurement (2 s interval). Data are represented as mean ± SEM (n = 3 experiments per condition). (F) Representative circular dichroism spectra of sodium phosphate buffer, liposomes only, 14 μM IDR^FBP17L^, and IDR^FBP17L^ incubated with liposomes (≤350 nm diameter) at 1:1 mole ratio at room temperature.

Increased curvature sensing for the IDR^FBP17L^ may be due to slower off-rate and longer dwell time on curved membrane. To test it, we compared the dynamics of membrane binding of IDR^FBP17L^ and F-BAR by fluorescence recovery after photobleaching (FRAP) experiment. After a single nanobar area within a circle area of 5 μm diameter was bleached, the lipid bilayer quickly recovered with a half-time of about 9 s (Figures 6A and 6E), indicating good membrane continuity and fluidity. Within the same 2-min window of the experiment, F-BAR, however, gave no detectable recovery (Figures 6B and 6E). It is consistent with the previous report that F-BAR domain tends to oligomerize and form higher-order assembly on membrane (Frost et al., 2008). In contrast, IDR^FBP17L^ showed significant recovery with a half-time of about 35 s (Figures 6C and 6E). To further test the source of recovery, we washed away the unbound IDR^FBP17L^ with phosphate-buffered saline and performed the same FRAP experiment. In this case, no recovery was observed at the same timescale (Figures 6D and 6E), suggesting that the recovery was mainly contributed by the exchange of IDR^FBP17L^ molecules from the solution rather than lateral diffusion of unbleached ones.

### Polyproline (PolyP) Region of IDR^FBP17L^ Is Involved More in Membrane Binding Than Curvature Sensing

Previously, it was shown that the disordered ALPS could sense curvature by forming stable helix upon binding of the lipid bilayer (Drin et al., 2007). However, for IDR^FBP17L^, no folded structure was detected by circular dichroism measurement even after binding to liposomes (Figure 6F), excluding the formation of alpha-helix or beta-sheet upon membrane binding. Nevertheless, we cannot rule out the possibility of hydrophobic insertion at this stage. Recently, it was found that PolyP helix had potential to transverse lipid bilayer (Franz et al., 2016; Kubyshkin et al., 2018). IDR^FBP17L^ contained six repeating proline residues (aa 340–345). Therefore, we investigated whether it influenced IDR^FBP17L^'s curvature sensing by substituting the first, third, and fifth prolines with alanine. The polyP mutant (IDR-APAPAP) showed weaker binding on the nanobar-lipid bilayer system when compared with wild-type IDR^FBP17L^ (Figures S5A and S5B) but similar curvature preferences (unpaired t test, p > 0.05) (Figure S5C). These data suggest that IDR^FBP17L^ is indeed unstructured, and the PolyP is beneficial for membrane binding but not essential for curvature sensing.

### High Concentration Promotes Curvature Sensing of IDR^FBP17L^

**Figure 7:**
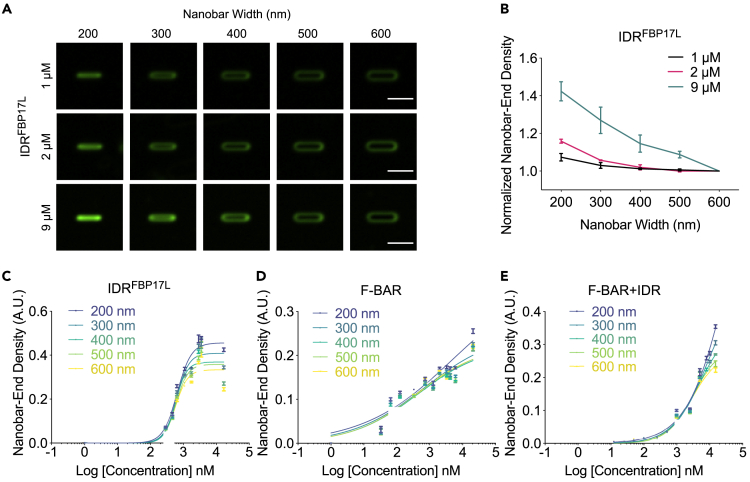
High Concentration Enhanced the IDR^FBP17L^'s Curvature Sensitivity (A) Averaged images of the IDR^FBP17L^ at different concentrations on nanobars with gradient width from 200 to 600 nm. Each nanobar image was averaged from over 60 nanobars. Scale bar: 2 μm. (B) Normalized nanobar-end density of IDR^FBP17L^ at different concentrations. Each point represents mean ± SEM from over 60 nanobars. (C–E) Plot of nanobar-end density of the IDR^FBP17L^ (C), F-BAR (D), and F-BAR + IDR (E) as a function of concentration. Lines are binding curves fitted with the Hill equation. Data are represented as mean ± SEM.

Curvature sensing had been shown to either increase (Ramamurthi et al., 2009) or decrease (Ramesh et al., 2013; Zeno et al., 2018) with higher concentration, indicating different dependency of curvature sensing on protein proximity. To further dissect this interaction, we compared the nanobar binding of IDR^FBP17L^ at a series of concentrations. We found that higher concentration led to more binding, and this effect became stronger on smaller nanobars (Figure 7A). When we plotted the nanobar-end density versus nanobar width in different concentrations, it clearly confirmed that curvature sensing was stronger at higher concentration (Figure 7B). Consistent with curvature sensing enhanced by protein proximity, binding curve of IDR^FBP17L^ on membranes showed stronger cooperativity compared with that of F-BAR or F-BAR + IDR (Figures 7C–7E). We could not reach saturating concentration for F-BAR or F-BAR + IDR, but for IDR^FBP17L^, we fitted its binding curves with the Hill equation and obtained *K*_*D*_ (0.60 ± 0.01 μM, mean ± SEM) and Hill coefficient (*H*) between 2 and 3, suggesting an ultrasensitive binding of IDR^FBP17L^ to highly curved membrane sites.

## Discussion

In this work, we discovered a conserved IDR in FBP17L that affected the kinetics of protein recruitment to the sites of membrane bending in live cells. This motivated us to examine *in vitro* whether it could directly sense curvature. A direct role in curvature sensing could only be demonstrated through *in vitro* studies because lipid-binding modules and IDRs are likely subjected to additional regulations in cells. With the nanobar-based SLB assay, we found that both the IDR and F-BAR domain in FBP17L could sense curvature of 200 nm in diameter, with IDR^FBP17L^ displaying much stronger sensitivity in terms of higher binding density at curved ends.

Current understanding of the mechanism of membrane curvature sensing is predominantly through well-defined domain structures that have membrane insertion (e.g., ENTH domain, amphipathic helices), curved membrane-binding surfaces (e.g., BAR domain), or both (e.g., N-BAR domain) (Antonny, 2006; Baumgart et al., 2011; Qualmann et al., 2011). Curvature sensing by IDR has several precedents (Bigay et al., 2005; Davidson et al., 1998; Morton et al., 2013). IDRs were proposed to play an important role in endocytic progression (Ungewickell and Hinrichsen, 2007). Large IDRs (>400 aa) from endocytic proteins sense curvature by imposing limitation on the conformational entropy when artificially tethered to membrane (Zeno et al., 2018, 2019). The IDR of FBP17L reported here is conserved, much smaller (66 amino acids), and does not require additional anchors for its recruitment to the membrane. When compared with F-BAR domain from FBP17, it has stronger curvature sensitivity than F-BAR at the same concentration. As IDR^FBP17L^ can dynamically associate with membrane and mediate curvature sensing, it senses curvature as a collective effect, not dependent on molecular scaffold over a curved surface.

F-BAR domain was widely assumed to sense curvature due to its crescent shape, but the curvature sensitivity of F-BAR from FBP17 had not been positively demonstrated in the literature. The ability of FBP17 to generate tubule was compellingly demonstrated *in vitro* (Itoh et al., 2005; Takano et al., 2008; Tsujita et al., 2006), in cells overexpressing FBP17 (Tsujita et al., 2006, 2013), and more recently in cells by optogenetics (Jones et al., 2020). In particular, by introducing F-BAR domain alone in the absence of IDR^FBP17L^ (e.g., aa 1–288 from FBP17, Jones et al., 2020, or aa1–300 from FBP17, Tsujita et al., 2006) to the plasma membrane, F-BAR was clearly sufficient to generate tubules. This raises an intriguing question of the interpretation of these tubulation experiments if they do not imply curvature sensing. Tubulation occurs when the energetic cost of bending membrane is lower than what is gained from protein-lipid binding and protein oligomerization on the membrane. Therefore, if the membrane is pre-curved, it seems reasonable that F-BAR would be more likely to bind to it. One possibility is that the modest level of curvature sensing we observe is sufficient for tubulation. The level of curvature sensing by F-BAR may also be synergistic to binding of other signaling lipids (see Discussion below in the limitations session). An alternative but not mutually exclusive possibility is that tubulation is a result of more complex biophysical processes. The arguments relating curvature generation to sensing assume that membrane is inert. In physiological context, tubulation may involve other more complex scenarios related to lateral reorganization of lipid bilayer. For instance, F-BAR domain may generate membrane domains similar to the BAR domain protein Lsp1 (Zhao et al., 2013). Formation of membrane domain can serve as a driving force for membrane bending to reduce line tension (Baumgart et al., 2003). If F-BAR binding to the membrane can reduce membrane tension (which remains to be experimentally tested), it is also possible that membrane will readily tubulate even without any curvature sensing of F-BAR. Finally, F-BAR domain of FBP17 interacts with membrane in two different conformations with its concave surface facing membrane or lying side way on the membrane (Frost et al., 2008). Gaussian curvature could play an important role in nucleating membrane tubulation (Noguchi, 2016, 2019), whereas our nanobar-based *in vitro* reconstitution assay does not control for the orientation of the F-BAR dimers.

We found that the sequence of recruitment of F-BAR proteins FBP17, CIP4, and the N-BAR proteins Endophilin and Amphiphysin in the cortical waves depends on their membrane-binding domains. Together with *in vitro* results confirming curvature sensing, we established that the sequential recruitment of these proteins in the traveling waves of mast cell cortex correlated with the progression of membrane curvature. A model of sequential curvature generation was hypothesized for endocytosis when the crystal structure of F-BAR was first solved (Shimada et al., 2007). It was thought that the F-BAR domain could sculpt a flat membrane into shallow curvature, followed by BAR protein, leading to the formation of clathrin-coated buds. Although it is an intuitive model, it conflicts with observations that F-BAR proteins of the FBP17/CIP4/Toca subfamily are recruited at late stages of clathrin-mediated endocytosis (Taylor et al., 2011), and its contribution to endocytosis is likely morphologically distinct from the maturation of clathrin-coated pits (Wu et al., 2010; Yang et al., 2017). Here we confirm that the sequential curvature generation model can be true but in a modified context from the original proposal. The dynamics of curvature generation covers a time period of about 8 s (F-BAR^FCHo1^ at −3 s, FBP17 or F-BAR^FBP17^ at 0 s, Endophilin, N-BAR^Endo^, or N-BAR^Amph^ at +5 s), which is much shorter than the time it takes to assemble an endocytic pit (30 s–1 min), and it corresponds to the late stage of the endocytosis. The rapid timescale is consistent with our previous characterization of membrane undulation using surface reflective interference contrast (SRIC) microscopy. As membrane waves propagate and oscillate with a period of 20–30 s, the lifetime of FBP17 puncta is about 8 s and the rise phase of SRIC lasts around 10 s (Wu et al., 2018). Although the phase differences are on the second timescale, it represents a significant fraction of the total time for membrane bending occurring in a living cell. Because of the timescale of this process, changes in bulk cytoplasmic protein concentrations can be minimal. Under these experimental conditions, we show that curvature preferences as defined by the end result of curvature generation *in vitro* can be reflected as the kinetics of their recruitments, suggesting that curvature-dependent membrane waves represent a unique context to infer curvature sensing.

### Limitations of the Study

Physiological contexts likely involve complex changes in the biochemical, geometrical, or mechanical properties of the membrane. Our *in vitro* assay aims to dissect a single factor (membrane curvature) and its effect on membrane binding without the compounding effects of membrane tension. Future study on the curvature sensing of the IDR^FBP17L^ may investigate the roles of phosphatidylinositide (PI) lipids, such as PI(4,5)P_2_ and PI(3,4,5)P_3_, both of which cycle in the similar phases as FBP17 in cortical waves and are likely physiological relevant (Xiong et al., 2016). Previous *in vitro* studies did not observe much difference in the binding of FBP17 to PS containing bilayer or PI(4,5)P_2_-containing bilayer (Itoh et al., 2005; Tsujita et al., 2006), but curvature-sensing effects may still be synergistic.

It also remains to be determined whether fine-tuning of the curvature sensing and recruitment kinetics of F-BAR domain-containing proteins through the IDR^FBP17L^ will result in cellular level functional consequences. Because IDR^FBP17L^ is precisely what defines the divergence of naturally occurring splicing isoforms, and its sequence is highly conserved across species, we speculate that its curvature sensing has functional implications. Interestingly, a recent work showed FBP17 isoform-specific function in neuronal morphology as well as an IDR-dependent membrane tubulation activity in cells. The IDR domain of FBP17 was found to be more potent in inducing neurite formation (Taylor et al., 2019). Consistent with our findings, such property of IDR of FBP17L is not shared by the IDR in CIP4. In addition, the IDR of the cytokinetic F-BAR protein Cdc15 in fission yeast was shown to be important for the maintenance of cytokinetic ring (Mangione et al., 2019). Cell protrusions and leading edge dynamics as well as cytokinesis are often simplified as actin cytoskeletal rearrangement but they are also controlled by cortical propagating waves and interlinked oscillatory networks (Bement et al., 2015; Bolado-Carrancio et al., 2020; Katsuno et al., 2015; Miao et al., 2017; Ruthel and Banker, 1999; Xiao et al., 2017). An intriguing possibility is that kinetic parameter changes could have implications in the conversion between regimes of dynamical systems (also known as bifurcations), which then result in morphogenetic outcomes. Information obtained here therefore would be expected to contribute to insights toward building a holistic model of cellular dynamics.

### Resource Availability

#### Lead Contact

Further information should be directed to Min Wu (wu.min@yale.edu).

#### Materials Availability

All materials generated from this study as listed in Transparent Methods are available. Requests should be directed to and will be fulfilled by the corresponding authors.

#### Data and Code Availability

Raw data and custom code are available upon reasonable requests.

## Methods

All methods can be found in the accompanying Transparent Methods supplemental file.

## Supplemental Information

Document S1. Transparent Methods and Figures S1–S5

## References

[bib1] Antonny B. (2006). Membrane deformation by protein coats. Curr. Opin. Cell Biol..

[bib2] Antonny B. (2011). Mechanisms of membrane curvature sensing. Annu. Rev. Biochem..

[bib3] Baumgart T., Hess S.T., Webb W.W. (2003). Imaging coexisting fluid domains in biomembrane models coupling curvature and line tension. Nature.

[bib4] Baumgart T., Capraro B.R., Zhu C., Das S.L. (2011). Thermodynamics and mechanics of membrane curvature generation and sensing by proteins and lipids. Annu. Rev. Phys. Chem..

[bib5] Bement W.M., Leda M., Moe A.M., Kita A.M., Larson M.E., Golding A.E., Pfeuti C., Su K.-C., Miller A.L., Goryachev A.B. (2015). Activator–inhibitor coupling between Rho signalling and actin assembly makes the cell cortex an excitable medium. Nat. Cell Biol..

[bib6] Bhatia V.K., Madsen K.L., Bolinger P.-Y.Y., Kunding A., Hedegård P., Gether U., Stamou D., Hedegard P., Gether U., Stamou D. (2009). Amphipathic motifs in BAR domains are essential for membrane curvature sensing. EMBO J..

[bib7] Bhatia V.K., Hatzakis N.S., Stamou D. (2010). A unifying mechanism accounts for sensing of membrane curvature by BAR domains, amphipathic helices and membrane-anchored proteins. Semin. Cell Dev. Biol..

[bib8] Bigay J., Casella J.-F., Drin G., Mesmin B., Antonny B. (2005). ArfGAP1 responds to membrane curvature through the folding of a lipid packing sensor motif. EMBO J..

[bib9] Bolado-Carrancio A., Rukhlenko O.S., Nikonova E., Tsyganov M.A., Wheeler A., Garcia-Munoz A., Kolch W., von Kriegsheim A., Kholodenko B.N. (2020). Periodic propagating waves coordinate RhoGTPase network dynamics at the leading and trailing edges during cell migration. Elife.

[bib10] Crooks G.E., Hon G., Chandonia J., Brenner S.E. (2004). WebLogo: a sequence logo generator. Genome Res..

[bib11] Davidson W.S., Jonas A., Clayton D.F., George J.M. (1998). Stabilization of α-Synuclein secondary structure upon binding to synthetic membranes. J. Biol. Chem..

[bib12] Van Der Lee R., Buljan M., Lang B., Weatheritt R.J., Daughdrill G.W., Dunker A.K., Fuxreiter M., Gough J., Gsponer J., Jones D.T. (2014). Classification of intrinsically disordered regions and proteins. Chem. Rev..

[bib64] Drin G., Casella J.F., Gautier R., Boehmer T., Schwartz T.U., Antonny B. (2007). A general amphipathic α-helical motif for sensing membrane curvature. Nat. Struct. Mol. Biol.

[bib13] Farsad K., De Camilli P. (2003). Mechanisms of membrane deformation. Curr. Opin. Cell Biol..

[bib14] Franz J., Lelle M., Peneva K., Bonn M., Weidner T. (2016). SAP(E) - a cell-penetrating polyproline helix at lipid interfaces. Biochim. Biophys. Acta.

[bib15] Frost A., Perera R., Roux A.A., Spasov K., Destaing O., Egelman E.H., De Camilli P., Unger V.M. (2008). Structural basis of membrane invagination by F-bar domains. Cell.

[bib16] Frost A., Unger V.M., De Camilli P. (2009). The BAR domain superfamily: membrane-molding macromolecules. Cell.

[bib17] Henne W.M., Kent H.M., Ford M.G.J., Hegde B.G., Daumke O., Butler P.J.G., Mittal R., Langen R., Evans P.R., McMahon H.T. (2007). Structure and analysis of FCHo2 F-bar domain: a dimerizing and membrane recruitment module that effects membrane curvature. Structure.

[bib18] Itoh T., Erdmann K.S., Roux A., Habermann B., Werner H., De Camilli P. (2005). Dynamin and the actin cytoskeleton cooperatively regulate plasma membrane invagination by BAR and F-bar proteins. Dev. Cell.

[bib19] Jones T., Liu A., Cui B. (2020). Light-inducible generation of membrane curvature in live cells with engineered BAR domain proteins. ACS Synth. Biol..

[bib20] Kakimoto T., Katoh H., Negishi M. (2004). Identification of splicing variants of Rapostlin, a novel Rnd2 effector that interacts with neural wiskott-aldrich syndrome protein and induces neurite branching. J. Biol. Chem..

[bib21] Katsuno H., Toriyama M., Hosokawa Y., Mizuno K., Ikeda K., Sakumura Y., Inagaki N. (2015). Actin migration driven by directional assembly and disassembly of membrane-anchored actin filaments. Cell Rep..

[bib22] Kozlov M.M., Campelo F., Liska N., Chernomordik L.V., Marrink S.J., McMahon H.T. (2014). Mechanisms shaping cell membranes. Curr. Opin. Cell Biol..

[bib23] Kubyshkin V., Grage S.L., Bürck J., Ulrich A.S., Budisa N. (2018). Transmembrane polyproline helix. J. Phys. Chem. Lett..

[bib24] Li X., Matino L., Zhang W., Klausen L., McGuire A.F., Lubrano C., Zhao W., Santoro F., Cui B. (2019). A nanostructure platform for live-cell manipulation of membrane curvature. Nat. Protoc..

[bib25] Madsen K.L., Bhatia V.K., Gether U., Stamou D. (2010). BAR domains, amphipathic helices and membrane-anchored proteins use the same mechanism to sense membrane curvature. FEBS Lett..

[bib26] Mangione M.S.C., Snider C.E., Gould K.L. (2019). The intrinsically disordered region of the cytokinetic F-BAR protein Cdc15 performs a unique essential function in maintenance of cytokinetic ring integrity. Mol. Biol. Cell.

[bib27] McDonald N.A., Takizawa Y., Feoktistova A., Xu P., Ohi M.D., Vander Kooi C.W., Gould K.L. (2016). The tubulation activity of a fission yeast F-bar protein is dispensable for its function in cytokinesis. Cell Rep..

[bib28] McMahon H.T., Gallop J.L. (2005). Membrane curvature and mechanisms of dynamic cell membrane remodelling. Nature.

[bib29] Miao Y., Bhattacharya S., Edwards M., Cai H., Inoue T., Iglesias P.A., Devreotes P.N. (2017). Altering the threshold of an excitable signal transduction network changes cell migratory modes. Nat. Cell Biol..

[bib30] Mim C., Cui H., Gawronski-Salerno J.A., Frost A., Lyman E., Voth G.A., Unger V.M. (2012). Structural basis of membrane bending by the N-BAR protein endophilin. Cell.

[bib31] Morton L.A., Yang H., Saludes J.P., Fiorini Z., Beninson L., Chapman E.R., Fleshner M., Xue D., Yin H. (2013). MARCKS-ED peptide as a curvature and lipid sensor. ACS Chem. Biol..

[bib32] Noguchi H. (2016). Membrane tubule formation by banana-shaped proteins with or without transient network structure. Sci. Rep..

[bib33] Noguchi H. (2019). Shape transition from elliptical to cylindrical membrane tubes induced by chiral crescent-shaped protein rods. Sci. Rep..

[bib34] Peter B.J. (2004). BAR domains as sensors of membrane curvature: the Amphiphysin BAR structure. Science.

[bib35] Pranke I.M., Morello V., Bigay J., Gibson K., Verbavatz J.M., Antonny B., Jackson C.L. (2011). α-Synuclein and ALPS motifs are membrane curvature sensors whose contrasting chemistry mediates selective vesicle binding. J. Cell Biol..

[bib36] Qualmann B., Koch D., Kessels M.M. (2011). Let’s go bananas: Revisiting the endocytic BAR code. EMBO J..

[bib37] Ramamurthi K.S., Lecuyer S., Stone H.A., Losick R. (2009). Geometric cue for protein localization in a bacterium. Science.

[bib38] Ramesh P., Baroji Y.F., Reihani S.N.S., Stamou D., Oddershede L.B., Bendix P.M., Nader S., Stamou D., Oddershede L.B., Bendix P.M. (2013). FBAR Syndapin 1 recognizes and stabilizes highly curved tubular membranes in a concentration dependent manner. Sci. Rep..

[bib39] Ruthel G., Banker G. (1999). Role of moving growth cone-like “wave” structures in the outgrowth of cultured hippocampal axons and dendrites. J. Neurobiol..

[bib40] Shimada A., Niwa H., Tsujita K., Suetsugu S., Nitta K., Hanawa-Suetsugu K., Akasaka R., Nishino Y., Toyama M., Chen L. (2007). Curved EFC/F-BAR-Domain dimers are joined end to end into a filament for membrane invagination in endocytosis. Cell.

[bib41] Simunovic M., Voth G.A., Callan-Jones A., Bassereau P. (2015). When physics takes over: BAR proteins and membrane curvature. Trends Cell Biol..

[bib42] Simunovic M., Evergren E., Golushko I., Prévost C., Renard H.F., Johannes L., McMahon H.T., Lorman V., Voth G.A., Bassereau P. (2016). How curvature-generating proteins build scaffolds on membrane nanotubes. Proc. Natl. Acad. Sci. U S A.

[bib43] Simunovic M., Bassereau P., Voth G.A. (2018). Organizing membrane-curving proteins: the emerging dynamical picture. Curr. Opin. Struct. Biol..

[bib44] Suetsugu S., Gautreau A. (2012). Synergistic BAR-NPF interactions in actin-driven membrane remodeling. Trends Cell Biol..

[bib45] Suetsugu S., Kurisu S., Takenawa T. (2014). Dynamic shaping of cellular membranes by phospholipids and membrane-deforming proteins. Physiol. Rev..

[bib46] Takano K., Toyooka K., Suetsugu S. (2008). EFC/F-BAR proteins and the N-WASP–WIP complex induce membrane curvature-dependent actin polymerization. EMBO J..

[bib47] Taylor M.J., Perrais D., Merrifield C.J. (2011). A high precision survey of the molecular dynamics of mammalian clathrin-mediated endocytosis. PLoS Biol..

[bib48] Taylor K.L., Taylor R.J., Richters K.E., Huynh B., Carrington J., McDermott M.E., Wilson R.L., Dent E.W. (2019). Opposing functions of F-BAR proteins in neuronal membrane protrusion, tubule formation, and neurite outgrowth. Life Sci. Alliance.

[bib49] Tsujita K., Suetsugu S., Sasaki N., Furutani M., Oikawa T., Takenawa T. (2006). Coordination between the actin cytoskeleton and membrane deformation by a novel membrane tubulation domain of PCH proteins is involved in endocytosis. J. Cell Biol..

[bib50] Tsujita K., Kondo A., Kurisu S., Hasegawa J., Itoh T., Takenawa T. (2013). Antagonistic regulation of F-BAR protein assemblies controls actin polymerization during podosome formation. J. Cell Sci..

[bib51] Ungewickell E.J., Hinrichsen L. (2007). Endocytosis: clathrin-mediated membrane budding. Curr. Opin. Cell Biol..

[bib52] Wu M., Huang B., Graham M., Raimondi A., Heuser J.E., Zhuang X., De Camilli P. (2010). Coupling between clathrin-dependent endocytic budding and F-BAR-dependent tubulation in a cell-free system. Nat. Cell Biol..

[bib53] Wu M., Wu X., De Camilli P. (2013). Calcium oscillations-coupled conversion of actin travelling waves to standing oscillations. Proc. Natl. Acad. Sci. U S A.

[bib54] Wu Z., Su M., Tong C., Wu M., Liu J. (2018). Membrane shape-mediated wave propagation of cortical protein dynamics. Nat. Commun..

[bib55] Xiao S., Tong C., Yang Y., Wu M. (2017). Mitotic cortical waves predict future division sites by encoding positional and size information. Dev. Cell.

[bib56] Xiong D., Xiao S., Guo S., Lin Q., Nakatsu F., Wu M. (2016). Frequency and amplitude control of cortical oscillations by phosphoinositide waves. Nat. Chem. Biol..

[bib57] Yamamoto H., Kondo A., Itoh T. (2018). A curvature-dependent membrane binding by tyrosine kinase Fer involves an intrinsically disordered region. Biochem. Biophys. Res. Commun..

[bib58] Yang Y., Xiong D., Pipathsouk A., Weiner O.D., Wu M. (2017). Clathrin assembly defines the onset and geometry of cortical patterning. Dev. Cell.

[bib59] Zeno W.F., Baul U., Snead W.T., DeGroot A.C.M.M., Wang L., Lafer E.M., Thirumalai D., Stachowiak J.C. (2018). Synergy between intrinsically disordered domains and structured proteins amplifies membrane curvature sensing. Nat. Commun..

[bib60] Zeno W.F., Snead W.T., Thatte A.S., Stachowiak J.C. (2019). Structured and intrinsically disordered domains within Amphiphysin1 work together to sense and drive membrane curvature. Soft Matter..

[bib61] Zhao H., Michelot A., Koskela E.V., Tkach V., Stamou D., Drubin D.G., Lappalainen P. (2013). Membrane-sculpting BAR domains generate stable lipid microdomains. Cell Rep..

[bib62] Zhao W., Hanson L., Lou H.-Y.Y., Akamatsu M., Chowdary P.D., Santoro F., Marks J.R., Grassart A., Drubin D.G., Cui Y. (2017). Nanoscale manipulation of membrane curvature for probing endocytosis in live cells. Nat. Nanotechnol..

[bib63] Zimmerberg J., Kozlov M.M. (2006). How proteins produce cellular membrane curvature. Nat. Rev. Mol. Cell Biol..

