# Supplementary material for: Comparative Study of Curvature Sensing Mediated by F-BAR and an Intrinsically Disordered Region of FBP17
Source: iScience. 2020 Oct 20;23(11):101712. doi: 10.1016/j.isci.2020.101712 (PMC7649350; doi:10.1016/j.isci.2020.101712)
Supplement: Document S1. Transparent Methods and Figures S1–S5 [file mmc1.pdf]

iScience, Volume *23*

Supplemental Information

Comparative Study of Curvature Sensing Mediated by F-BAR and an Intrinsically Disordered Region of FBP17

Maohan Su, Yinyin Zhuang, Xinwen Miao, Yongpeng Zeng, Weibo Gao, Wenting Zhao, and Min Wu

Supplemental Figures

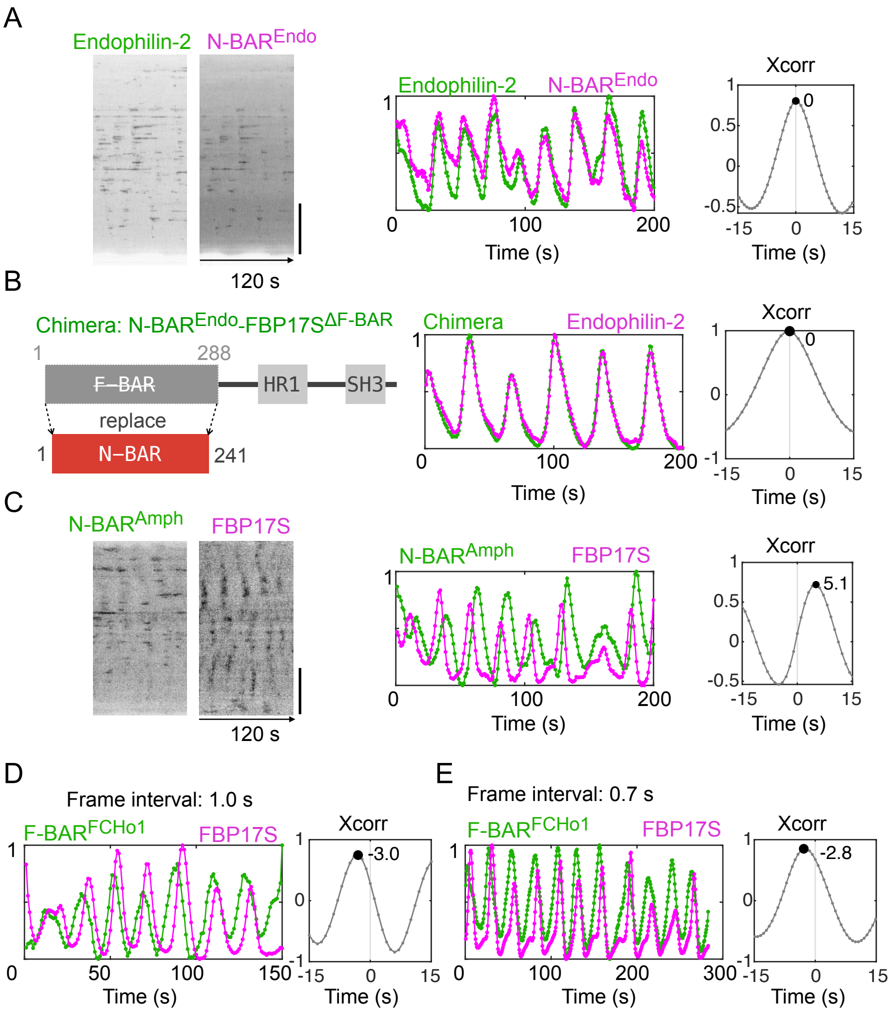

Figure S1. Phase of recruitment in cortical waves. Related to Figure 1.

A. Kymographs of the same line within cell area (**left**), intensity profile of the same ~2x2 μm^2 region-of-interest (ROI) in the cell (**middle**), and cross-correlation (**right**) of waves of Endophilin-2-GFP and N-BAR^{Endo} (Endophilin-1 a.a. 1-247)-mCherry. Time resolution: 0.7 s. Scale bar: 10 μm.

B. Left: Diagram of domain structure of chimeric N-BAR^{Endo}-FBP17S$^{\Delta F\text{-}BAR}$-GFP (not drawn to scale). Intensity profile (**middle**), and cross-correlation (**right**) of waves of it relative to waves of Endophilin-2-mCherry. Time resolution: 1.0 s.

C. Kymographs (**left**), intensity profile (**middle**) and cross-correlation (**right**) of waves of N-BAR^{Amph}(Amphiphysin-1 a.a. 1-236)-GFP and FBP17-mCherry. Time resolution: 1.0 s. Scale bar: 10 μm.

D, E. Phase shift of F-BAR^{FCHo1} (FCHo1 a.a. 1-275)-GFP relative to mCherry-FBP17S imaged at time interval of 1.0 s (**D**) and 0.7 s (**E**). **Left**: Intensity profile. **Right:** Cross-correlation of GFP relative to mCherry.

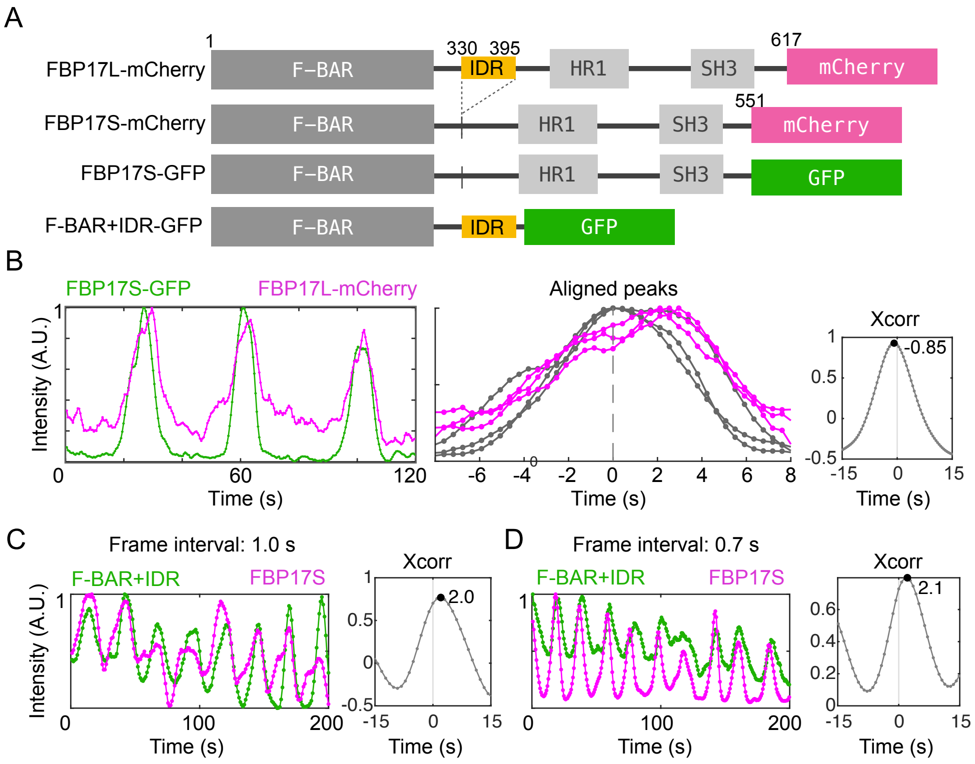

Figure S2. Phase differences of FBP17 isoforms and mutants in travelling waves. Related to Figure 2.

A. Diagram of domain structures of C-terminal tagged long isoform FBP17L, short isoform FBP17S, and F-BAR+IDR (FBP17L a.a. 1-395) used in this figure. The intrinsic disordered region (IDR) is shown in yellow.

A. Intensity profile of waves of co-expressed FBP17S-GFP and FBP17L-mCherry (**left**), aligned peaks of GFP channel (**bottom**), and cross-correlation of GFP relative to mCherry.

C, D. The phase lag of F-BAR+IDR-GFP relative to FBP17S-mCherry imaged at time interval of 1.0 s (**C**) and 0.7 s (**D**). **Left**: Intensity profile. **Right:** Cross-correlation of GFP relative to mCherry.

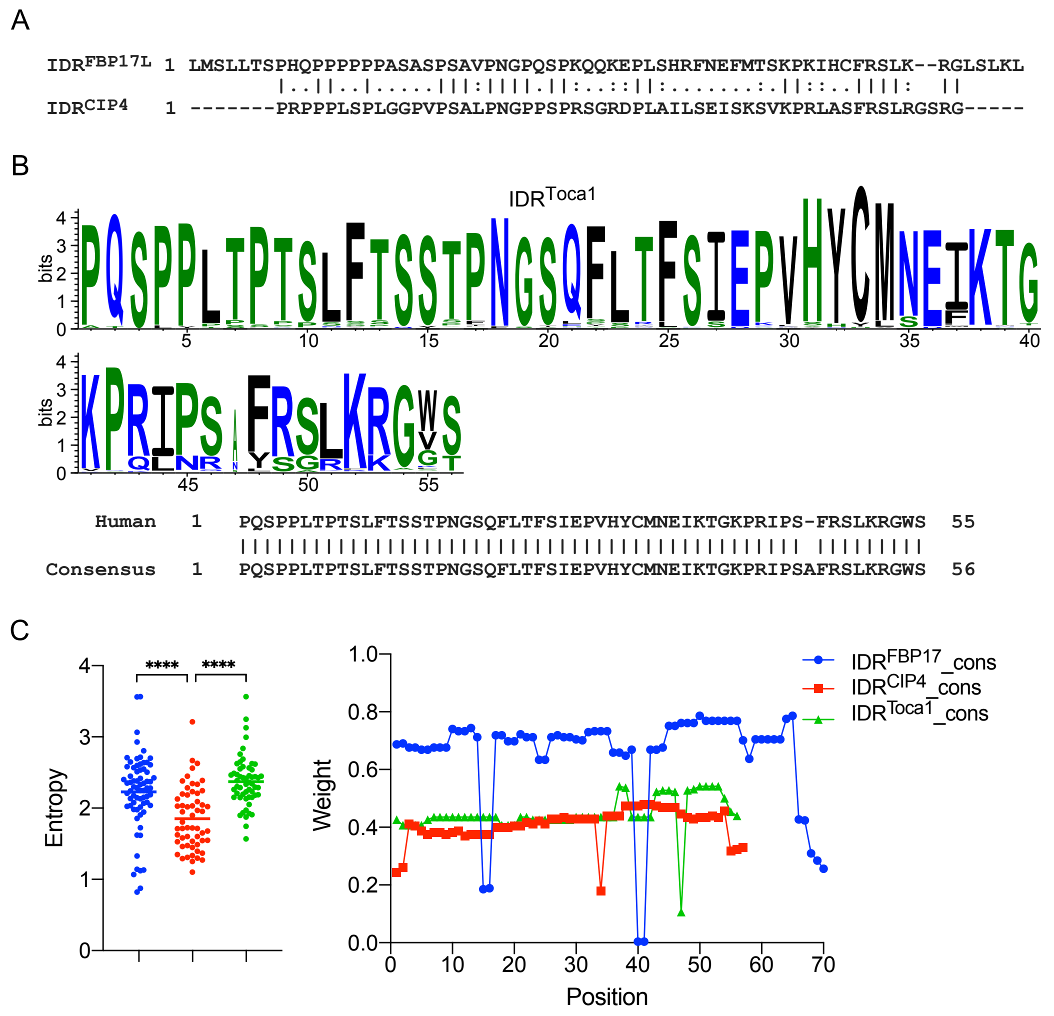

Figure S3. Differences between IDRs from FBP17, Toca1 and CIP4. Related to Figure 3.

A. equence alignment of IDRs of human FBP17L and CIP4.

B. **Top:** WebLogo of the IDR of Toca1 from all 299 species that have it. **Bottom**: Sequence alignment of the IDR from human FBP17L and the consensus sequence from WebLogo 3.

C. Sequence conservation (entropy) and splicing frequency (weight) of IDRs from FBP17, CIP4 and Toca1. Mann–Whitney U test: **** denotes $P<0.0001$.

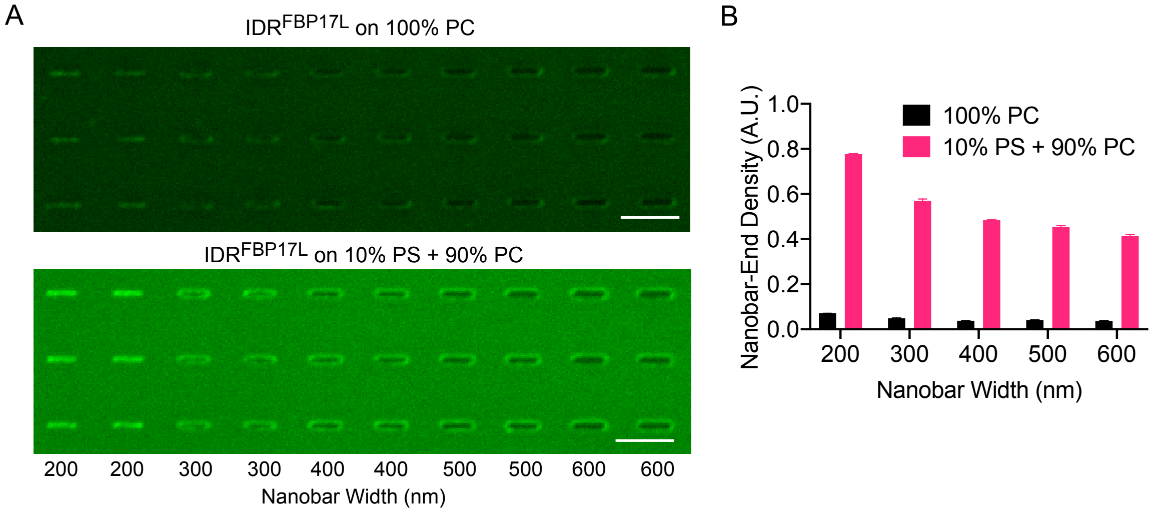

Figure S4. Membrane binding of IDR^{FBP17L} on phosphatidylcholine (PC)-coated and phosphatidylserine (PS)-coated nanobars. Related to Figure 4.

A. Confocal images of 9 µM Alexa Fluor 647 labelled IDR^{FBP17L} on (**top**) 100% egg PC-coated gradient nanobars and (**bottom**) 10% brain PS + 90% egg PC-coated gradients nanobars. Scale bar: 5 µm.

B. Nanobar-end density of 9 µM IDR^{FBP17L} on 100% egg PC-coated gradient nanobars and 10% brain PS + 90% egg PC-coated gradients nanobars. Each point represents mean from over 100 nanobars and the error bar represents standard error of the mean.

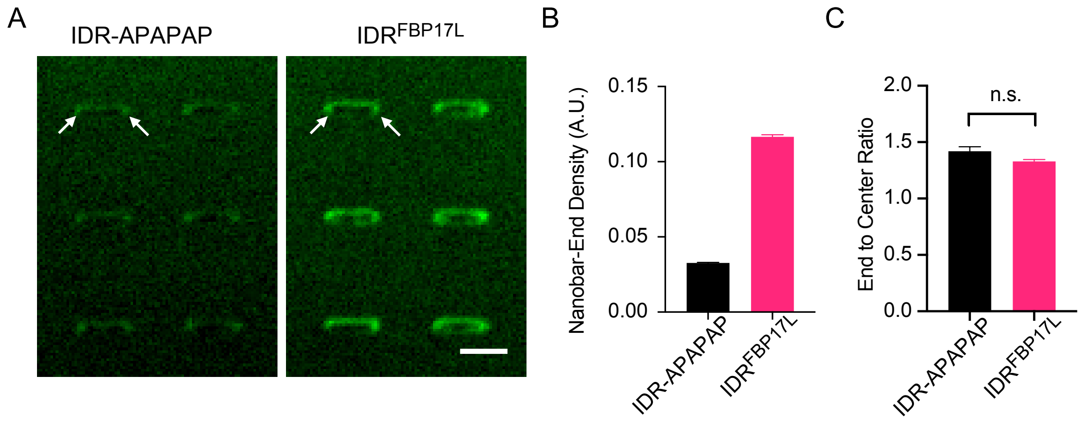

Figure S5. Curvature sensing of IDR-polyproline mutant (IDR–APAPAP) on nanobar arrays. Related to Figure 6.

A. onfocal images of 9 μM Alexa Fluor 647 labelled IDR-APAPAP (**left**) and IDR^{FBP17L} (**right**) on 10% brain PS + 90% egg PC-coated nanobars of 300 nm width. Arrowheads indicate two nanobar-ends. Scale bar: 2 μm.

B. Nanobar-end density of 9 μM IDR-APAPAP and IDR^{FBP17L} on 100 nanobars of 300 nm width. Error bar represents standard error of the mean.

C. End to center ratio of IDR-APAPAP and IDR^{FBP17L} on 100 nanobars of 300 nm width. Unpaired t-test with Welch's correction: ns: p>0.05. Error bar represents standard error of the mean.

Transparent Methods

Materials

L-α-phosphatidylcholine (Egg PC, Chicken, 840051) and L-α-phosphatidylserine (Brain PS, Porcine, 840032) were from Avanti Polar Lipids, Inc. Texas Red 1,2-Dihexadecanoyl-sn-Glycero-3-Phosphoethanolamine, Triethylammonium Salt (Texas Red DHPE) was from Invitrogen.

Plasmids

mCherry-FBP17L, FBP17S-EGFP were from Pietro De Camilli lab. Chimeric N-BAR^{Endo}-FBP17S$^{\Delta F\text{-}BAR}$-GFP were generated by replacing F-BAR domain (a.a. 8–288) of FBP17S-EGFP with BAR domain (a.a. 1–241) of rat Endophilin2 (Wu et al., 2018). EGFP-FBP17L and FBP17L-mCherry were acquired by cloning human FBP17L (UniProt ID: Q96RU3, full length, a.a. 1-617) from mCherry-FBP17L into pEGFP-C2 (XhoI/XbaI) and pmCherry-N1 (XhoI/EcoRI) vectors. FBP17L-EGFP was generated by adding a.a. 330-390 back through PCR-ligation to FBP17S-EGFP. EGFP-FBP17S and mCherry-FBP17S were generated by deleting a.a. 330-395 from EGFP-FBP17L and mCherry-FBP17L. FBP17S-mCherry was obtained by cloning FBP17S from FBP17S-EGFP into pmCherry-N (XhoI/EcoRI). To generate truncation mutants, F-BAR (a.a. 1-329) and F-BAR+IDR (a.a. 1-395) from human FBP17L were cloned into pEGFP-N1 (both by XhoI/BamHI). N-BAR (a.a. 1-247) from human Endophilin A1 were cloned into pmCherry-N1 (EcoRI/BamHI). N-BAR (a.a. 1-236) from human Amphiphysin1 was cloned into pEGFP-N1 (Xhol/EcoRI). F-BAR (a.a. 1-275) from mouse FCHo1 was cloned into pmCherry-N1 (EcoRI/KpnI). Point mutations (K66E and K166A) on F-BAR-EGFP were produced by Q5 Site-Directed Mutagenesis Kit (New England BioLabs). Endophilin-2-GFP was a gift from Pietro De Camilli lab. Identity of plasmids was confirmed by Sanger sequencing.

Cell Culture and Transfection

RBL-2H3 cells (tumor mast cells) were maintained in MEM medium with 20% FBS (Wu et al., 2018). Cells were harvested with TrypLE (Life Technologies) 2–5 days after passage. Electroporation with Neon transfection system (Life Technologies) was utilized for transient transfections. After transfection, cells were plated at subconfluent densities in 35-mm glass bottom dishes (Cellvis, CA) overnight. Cells were sensitized with mouse monoclonal anti-2,4-dinitrophenyl IgE (Sigma-Aldrich) at 0.5 $\mu g\, ml^{-1}$ overnight and stimulated with 80 $ng\, ml^{-1}$ multivalent antigen, 2,4-Dinitrophenylated bovine serum albumin (Life Technologies) 0.5 to 1 h before imaging. During imaging, the cells were either in medium or Tyrode's buffer (135 mM NaCl, 5.0 mM KCl, 1.8 mM $CaCl_2$, 1.0 mM $MgCl_2$, 5.6 mM glucose, and 20 mM HEPES, pH 7.4).

TIRF Imaging and Image Analysis

The TIRF microscopy imaging system was consisted of a Nikon Ti-E inverted microscope, iLAS2 motorized TIRF illuminator (Roper Scientific, Evry Cedex, France) and Prime 95B CMOS Camera (Teledyne Photometrics, AZ) (Wu et al., 2018). All images were acquired through a TIRF-CFI objective (Apochromat TIRF 100XH numerical aperture

(N.A.) 1.49, Nikon). For stream acquisition mode, the exposure time was set at 50 ms per channel to achieve 0.21 s frame interval (instead of 100 ms at 0.7 or 1.0 s interval). Images were analyzed by Fiji-ImageJ (Schindelin et al., 2012) to generate micrographs, kymographs. Plots and graphs were generated in MATLAB 2018b (Mathworks). Phase shift was calculated by custom code of cross-correlation in MATLAB as the following. A ~2×2 μm^2 ROI was selected at an area where waves were prominent and last for at least three cycles. The corresponding intensity profile versus time was smoothed (average span = 5 timepoints) and normalized to 0-1 range (the lowest/highest intensity as 0/1). Cross-correlations were calculated using the build-in function (xcorr). The phase shift was always extracted at the maximum cross-correlation. Only data of maximum cross-correlation > 0.6 was analyzed and shown.

Bioinformatics

The sequences of FBP17 and CIP4 from all species were extracted from UniProtKB by the search term 'gene exact:fnbp1' and 'gene_exact:trip10'. Sequence alignment was performed by Clustal Omega and the sequence logo with its entropy and weight data was generated by WebLogo 3. Intrinsic disorder prediction was performed by IUPred2A (Mészáros et al., 2018). The distribution of the IDR in the tree of life was produced by lineage and taxonomy report of Blastp (protein-protein BLAST) result using the human IDR sequence against database Non-redundant protein sequences. Each hit with a species had E value $<10^{-36}$.

Fabrication of Nanobar Chips

The nanobar arrays used in this study were fabricated on a square quartz coverslip by using electron-beam lithography (Zhao et al., 2017). In brief, 15×15×0.2 mm square quartz coverslips were spin-coated with positive electron-beam resist polymethyl methacrylate (MicroChem) with around 300 nm height. Conductive protective coating was applied later with AR-PC 5090.02 (Allresist). Nanobar patterns were drawn by Electron Beam Lithography (FEI Helios NanoLab) and developed in isopropanol: methyl isobutyl ketone = 3:1 solution. A 100 nm-height chromium mask was generated via thermal evaporation (UNIVEX 250 Benchtop) and lifted off in acetone. Nanobars were subsequently generated by reactive ion etching with the mixture of CF_4 and CHF_3 (Oxford Plasmalab80). Scanning Electron Microscopy (FEI Helios NanoLab) imaging was performed after 10 nm chromium coating to verify the dimensions of nanobars. Before usage, the nanobar chips were immersed in Chromium Etchant (Sigma-Aldrich) until the chromium masks were removed.

Peptide Synthesis and Protein Purification

The IDR of human FBP17L (a.a. 330-395, LMSLLTSPHQ PPPPPPASAS PSAVPNGPQS PKQQKEPLSH RFNEFMTSKP KIHCFRSLKR GLSLKL) (89% purity) was synthesized by Sangon Biotech (Shanghai) Co., Ltd. IDR of human CIP4 (a.a. 329-384+cysteine, PRPPPLSPLG GPVPSALPNG PPSPRSGRDP LAILSEISKS VKPRLASFRS LRGSRGC) (89% purity, additional cysteine was added at the C-terminus for labelling) was synthesized by GL Biochem (Shanghai) Ltd. The molecular weight was confirmed by HPLC and mass spectrometry.

Human F-BAR (a.a. 1-329) of FBP17L was cloned into pNIC-CTHF and expressed as a fusion protein with cleavable C-terminal His-Flag tag. Human F-BAR+IDR (a.a. 1-395) of FBP17L was cloned into pNIC-GST vector and expressed as fusion proteins with cleavable N-terminal GST-His tag. All proteins were expressed in *E.coli* BL21 (DE3) Rosetta T1R. The resuspended bacterial pellet was thawed and sonicated (Sonics Vibra-cell) at 70% amplitude, 3 s on/off for 3 min, on ice. The lysate was clarified by centrifugation at 47,000 g, 4 ºC for 25 min. The supernatant was then filtered through 1.2 µm syringe filters and loaded onto ÄKTA Xpress system (GE Healthcare).

The purification regime is briefly as follows. The supernatants were loaded on nickel-ion metal affinity chromatography (IMAC) columns. The columns were washed with wash 1 (20 mM HEPES, 500 mM NaCl, 10 mM Imidazole, 10 % (v/v) glycerol, 0.5 mM tris(2-carboxyethyl)phosphine (TCEP), pH 7.5) and wash 2 (20 mM HEPES, 500 mM NaCl, 25 mM Imidazole, 10 % (v/v) glycerol, 0.5 mM TCEP, pH 7.5) buffer respectively at 0.8 mL/min flow rate until a stable baseline was obtained respectively. The eluted proteins were then collected and stored in sample loops on the system and then injected into Gel Filtration columns. Elution peaks were collected in 2 mL fractions and analyzed on SDS-PAGE gels. The entire purification was performed at 4º C. All IMAC columns are stripped of Ni^{2+}, cleaned-in-place with 0.5 M NaOH, re-charged with Ni^{2+} (0.1 M $NiCl_2$) and stored in 20% ethanol in between purification runs. All GF columns are cleaned-in-place with 0.5 M NaOH and stored in 20% ethanol in between purification runs. Relevant peaks were pooled, TCEP was added to a total concentration of 2 mM. The protein sample was concentrated in Vivaspin 20 filter concentrators (VivaScience) at 15 ºC to approximately 15 mg/mL. Protein concentration was assessed by measuring absorbance at 280 nm on Nanodrop ND-1000 (Nano-Drop Technologies) and purity was assessed on SDS-PAGE gel. The final protein was in buffer with 20 mM HEPES pH 7.5, 300 mM NaCl, 10% (v/v) glycerol, 2 mM TCEP, then aliquoted, frozen in liquid nitrogen and stored at -80 ºC.

Protein Labelling

Alexa Fluor 647 C2 Maleimide (A20347, Invitrogen) was used to label the only cysteine of IDR^{FBP17L}. For IDR^{CIP4}, additional cysteine was added at the C-terminus during synthesis as mentioned in the protein purification section. Ligation was at room temperature for 30 min and 100 mM glutathione was added to stop the reaction. Unconjugated Maleimide dyes were dialyzed from labeled IDR^{FBP17L} using 3.5 kDa membrane in 1 L phosphate-buffered saline (PBS) overnight twice at 4°C. Amicon Ultra-0.5 mL Centrifugal Filters (Merck) were used to concentrate the labeled proteins. The final protein concentration and labeling ratio were measured using NanoDrop UV-Vis Spectrophotometer (Thermo Scientific).

Alexa Fluor 488 tetrafluorophenyl ester dyes (A37563, Invitrogen) were dissolved in DMSO at a concentration of 10 mg/ml and stored at -80 °C. F-BAR and F-BAR+IDR were diluted to 2 mg/ml in PBS. 1 M sodium bicarbonate solution was prepared and added to the protein solution at a final concentration of 0.1M (pH~8.3). 10 µl TFP ester dyes were warmed up to room temperature and transferred to the protein solution. The reaction mixture was inverted for 1 h at room temperature, resulting in a dye/protein labeling ratio between 1 and 1.5. Unconjugated TFP ester dyes

were separated from labeled proteins using HiTrap Desalting columns with Sephadex G-25 resin (GE Healthcare Life Sciences).

Preparation of Lipid Vesicles

The molar composition of lipid vesicles is egg PC mixed with 0.5 mol% of Texas Red-DHPE and 10 mol % of brain PS. Lipids were dissolved in chloroform and mixed at a fixed molar ratio as described above. The lipid mixture was dried down in a brown glass vial with 99.9% nitrogen gas for 5 min, followed by vacuum drying for 3 h to remove the remaining chloroform. The dried lipid film was resuspended in PBS at a concentration of 2 mg/ml and sonicated for 30 min. The lipid mixture was then transferred to a 1.5 ml tube and freeze-thawed for 15 times (20 s in liquid nitrogen and 2 min in 42 °C water bath). The lipid mixture was extruded through a polycarbonate membrane with 100 nm pore size using an extruder set with holder/heating block (610000-1EA, Sigma-Aldrich). The lipid vesicle solution was stored at 4 °C and used within 2 weeks.

Lipid Bilayer Formation on Nanobar Chips

The nanobar chips were cleaned with piranha solution (7 parts of concentrated sulfuric acid and 1 part of 30% hydrogen peroxide solution) overnight and washed with continuous stream of deionized water to remove the acids. Subsequently, the nanobar chips were dried in 99.9% nitrogen gas and cleaned with air plasma in a plasma cleaner (Harrick Plasma) for 30 min to remove any remaining impurities on the surfaces before lipid bilayer formation. The cleaned chips were attached with a polydimethylsiloxane (PDMS) chamber and lipid vesicles were loaded into the PDMS channel and incubated for 15 min to form the lipid bilayer. PBS was used to wash away the unbound vesicles in the PDMS channel. Protein solution was subsequently added onto the lipid-bilayer-coated nanobar and incubated for 5 min at room temperature before microscopy imaging.

Imaging of Protein-Nanobar Interaction and Quantification

Purified proteins distribution on lipid-bilayer-coated nanobar arrays were imaged with laser scanning confocal microscopy (Zeiss LSM 800 with Airyscan) using a 100× (N.A.1.4) oil objective, 1 Airy Unit=0.9 µm. Texas Red-DHPE-containing lipid bilayer was excited at 561 nm and detected at 570-645 nm with gain of 650 V. Alexa Fluor 488-labeled F-BAR and F-BAR+IDR were excited at 488 nm and detected at 490-570 nm with gain of 700 V. Alexa Fluor 647-labeled IDR^{FBP17L} was excited at 633 nm and detected at 645-700 nm with gain of 750 V. Each image was obtained at a scanning speed of 20 s/image (pixel dwell time 4 µs), averaging mode of 4 times/line, and has a resolution of 512×512 pixels, with a pixel size of 124 nm and 16-bit depth.

Image processing and analysis were performed using custom-written MATLAB (Mathworks) code adapted from previous study (Zhao et al., 2017). In brief, individual nanobar position in the lipid channel image was located using a square mask (51×51 pixels) centered at the nanobar, then same mask was applied to generate individual nanobar image of the protein channel. Background of each individual nanobar image was corrected by subtracting a local background image generated using meshgrid function in Matlab based on the four ROIs (15×15 pixels) at the corners

of the image. Background-corrected individual nanobar images with the same nanobar dimension were then averaged across different arrays and experimental repeats to generate averaged images (as shown in **Fig. 4F**). To quantify the signal at nanobar-end and nanobar-center, each nanobar was segmented into three ROIs (two nanobar-ends and a nanobar-center). The size of nanobar-end ROI was adjusted according to the dimension of nanobar to minimize the covering of the nearby non-curved center of nanobar. Intensity values of pixels within each ROI were then integrated. To compare lipid and protein channels, the intensities of background-corrected individual nanobar images were first normalized to the percentages of 600 nm nanobar-center intensity acquired in the same image. Then protein density at each nanobar was measured by the ratio of normalized protein intensity to normalized lipid intensity (data shown in **Fig. 5G**). The 3D surface plots of protein distribution on nanobar was obtained from averaged images by Fiji-ImageJ. Statistical analysis was performed by PRISM 8 (GraphPad).

Supplemental References

Mészáros, B., Erdös, G., and Dosztányi, Z. (2018). IUPred2A: Context-dependent prediction of protein disorder as a function of redox state and protein binding. Nucleic Acids Res. *46*, W329–W337.

Schindelin, J., Arganda-Carreras, I., Frise, E., Kaynig, V., Longair, M., Pietzsch, T., Preibisch, S., Rueden, C., Saalfeld, S., Schmid, B., et al. (2012). Fiji: an open-source platform for biological-image analysis. Nat. Methods *9*, 676–682.
